# A systematic review of the prediction of consumer preference using EEG measures and machine-learning in neuromarketing research

**DOI:** 10.1186/s40708-022-00175-3

**Published:** 2022-11-14

**Authors:** Adam Byrne, Emma Bonfiglio, Colin Rigby, Nicky Edelstyn

**Affiliations:** 1 Keele Business School, Denise Coates Foundation Building, Keele University, Staffordshire, ST5 5AA UK; 2 Faculty of Humanities and Social Sciences, Keele University, Staffordshire, UK; 3 Risual Limited, Staffordshire, UK; 4 School of Psychology, Keele University, Staffordshire, UK

**Keywords:** EEG, ERP, Neuromarketing, Machine learning, Systematic review, Time–frequency

## Abstract

**Introduction:**

The present paper discusses the findings of a systematic review of EEG measures in neuromarketing, identifying which EEG measures are the most robust predictor of customer preference in neuromarketing. The review investigated which TF effect (e.g., theta-band power), and ERP component (e.g., N400) was most consistently reflective of self-reported preference. Machine-learning prediction also investigated, along with the use of EEG when combined with physiological measures such as eye-tracking.

**Methods:**

Search terms ‘neuromarketing’ and ‘consumer neuroscience’ identified papers that used EEG measures. Publications were excluded if they were primarily written in a language other than English or were not published as journal articles (e.g., book chapters). 174 papers were included in the present review.

**Results:**

Frontal alpha asymmetry (FAA) was the most reliable TF signal of preference and was able to differentiate positive from negative consumer responses. Similarly, the late positive potential (LPP) was the most reliable ERP component, reflecting conscious emotional evaluation of products and advertising. However, there was limited consistency across papers, with each measure showing mixed results when related to preference and purchase behaviour.

**Conclusions and implications:**

FAA and the LPP were the most consistent markers of emotional responses to marketing stimuli, consumer preference and purchase intention. Predictive accuracy of FAA and the LPP was greatly improved through the use of machine-learning prediction, especially when combined with eye-tracking or facial expression analyses.

## Introduction

The present systematic review aimed to investigate the use of EEG measures in neuromarketing. We specifically focused on identifying which ERP and TF effects were most consistently associated with consumer preference and purchase intention, the best computational approaches to predict consumer behaviour, and which biometric measures are best combined with EEG measures to improve predictive accuracy.

Marketing is used to help a product inform, engage, and sustain its target audience by identifying and manipulating consumer preferences [167, 188, 288]. Conventional market research depends on self-report measures such as questionnaires, interviews, and focus-group discussions [167, 188, 288]. However, the traditional approach to marketing is usually only capable of conducting posteriori analysis of consumer preference towards marketing stimuli [288]. Self-report methods may also provide potentially unreliable or incomplete data due to participants misremembering their experiences or conforming to social desirability bias [288].

Neuromarketing overcomes the limitations of traditional marketing methods by capturing consumers’ unspoken cognitive and emotional responses to marketing stimuli using neuroimaging and biometric devices [11, 21, 30, 213, 304]. This allows for the concurrent recording of consumers’ emotional responses while engaging with marketing stimuli, and can detect emotional responses that consumers may be unwilling to report or may even be unware of [11, 21, 30, 213, 304]. The main techniques applied within this paradigm are neuroimaging measures, such as functional magnetic resonance imaging (fMRI) and electroencephalography (EEG); and biometric measures such as the galvanic skin response (GSR) and eye-tracking (ET) [163].

EEG measures and physiological measures (e.g., ET, GSR) are most commonly used in neuromarketing research due to them being relatively inexpensive and easy to implement [8, 130, 257]. EEG measurements are considered useful for their high temporal resolution and ability to adapt traditional experimental designs into neuroscience experiments [8, 130, 257], and are highly compatible with machine-learning algorithms due to the richness of data collected [59, 144, 164]. By comparison, physiological measures collect simpler data types, taken from bodily responses indexing changes in arousal, emotion and visual attention [18, 214, 221, 257, 290]. Heart-rate responses, GSR, and are typically used in neuromarketing stimuli to measure changes in physiological arousal in response to marketing stimuli, but cannot separate positive from negative responses [18, 214, 257]. Similarly, ET can be used to measure how marketing stimuli draw visual attention [10, 168, 221, 290] and facial expression analysis can be used to detect specific emotional responses to stimuli such as disgust or anger [60, 155, 187].

In contrast, neuroimaging measures such as fMRI are favoured due to their ability to accurately localise neural activity through detection of haemodynamic blood flow, but has poor temporal resolution when compared to EEG [134, 149, 224]. fMRI may also be less favourable in neuromarketing research than physiological and EEG measures due to the high cost associated with fMRI measures, and their lack of portability [134, 149, 224].

However, despite the current popularity of neuromarketing, it remains unclear which measures are the most effective and in which context they are best used. For example, while fMRI is often preferred for its spatial resolution and EEG is preferred for its temporal resolution [200], the comparative effectiveness of these measures in neuromarketing research is yet to be systematically investigated. Further, the data recorded by neuromarketing devices can be analysed in several ways and situations. For example, EEG data can be considered in terms of event-related potentials (ERPs) occurring milliseconds after the presentation of a stimulus [172, 184, 238], or in terms of changes in relative power in specified frequency bands [197, 256]. Recent developments in computer science have further allowed for machine-learning algorithms [2] to precisely predict consumer preference, purchasing behaviour, and remembered events [44, 51].

We, therefore, conducted a large-scale systematic review on the field of neuromarketing, with the express purpose of investigating the use of differing neuroimaging and biometric measures to determine their best use in the context of marketing research. To ease analysis, the systematic review was divided into three subsets based on broad neuromarketing measures: EEG measures, functional imaging measures, and biometric measures. The subset discussed presently focused on the use of EEG measures in neuromarketing. Specifically, the effectiveness of phase-locked and time–frequency (TF) analysis methods were compared, as well as sub-analysis methods within these fields (e.g., alpha- compared to theta-band activity). EEG combined with other neuromarketing measures (e.g., EEG combined with eye-tracking) was further investigated. Finally, the effectiveness of algorithmic approaches (e.g., machine-learning) to analysing EEG data were compared to traditional statistical methods.

The present article, therefore, focused its investigations on the following research questions:

- What different analysis methods are currently used for EEG research in neuromarketing?
- In which conditions are differing ERP components and TF effects significantly modulated?
- Which EEG effects are best used to predict consumer preference and emotional responses to marketing stimuli?
- In which ways are EEG measures best combined with other measures, and which measures are best combined?
- Does machine learning improve the predictive accuracy of EEG measures in neuromarketing?

The research questions were generated in descending order to first identify the TF and ERP measures currently used in neuromarketing research, and then to investigate which emotional processes each effect was most consistently associated with. Following the initial identification of relevant EEG measures, consumer preference was investigated, and the effects most consistently associated with preference and purchase intention were assessed. Finally, the use of other biometric measures (e.g., ET, GSR) when combined with EEG was investigated, as well as the best computational approaches to predict consumer preference and purchase intention (e.g., machine learning algorithms compared to regression analyses).

These questions will allow us to gain a comprehensive account of the use of EEG measures in neuromarketing, which components are the most useful, and in which situations they are best used. After the introduction, the systematic review methodology will be discussed, followed by the systematic review findings, and a broader discussion of these findings.

## Methods

A systematic literature review was performed to collect and assess the measures used in neuromarketing. This method was selected due to systematic literature reviews' high degree of objectivity and replicability [104]. A systematic review uses pre-defined methods to collect, select, and analyse collected literature, with the purpose of unbiased evidence collection to reach an impartial conclusion [135].

The present systematic review aimed to examine measures used in neuromarketing (i.e., neuroimaging and physiological measures) and identify the best use of these measures. To ease analysis, literature was subdivided into three categories; EEG measures, physiological measures, and functional imaging measures. The present paper represents the first subset of the systematic review; EEG measures. The analysis of the literature collected in this subset focused on comparing different EEG analysis approaches (e.g., TF and ERP analysis) in terms of their effectiveness when used in different kinds of neuromarketing research.

### Search terms

Studies were collected from relevant journals and databases using key search terms. The primary search terms were the simple terms' neuromarketing' and 'consumer neuroscience', intended to catch studies that self-identified as belonging to the field of neuromarketing. However, additional studies may have used neuroscience or physiological measures to investigate marketing-relevant behaviour without explicitly using these terms. For this reason, additional search terms were used to identify studies that used neuroscience or physiological measures (e.g., EEG, MRI, GSR, ET) combined with marketing or consumer investigation.

The key search terms were used as selection criteria for the titles, keywords, abstracts, and body of text in the selected databases and journals. Document types included in the search were ‘articles’, and time limits were not established. Therefore, the initial search resulted in a shortlist of relevant publications to be considered for inclusion in the review. Duplicate articles were excluded from subsequent analysis.

To ensure the maximum number of neuromarketing studies were collected, literature searches were conducted in multiple databases and individual journals. Databases were selected from those that are internationally recognised and widely used as a source of research for distinct post-graduate programmes. Table 1 presents the database sources and the search terms used, and the number of articles found within each database.

**Table 1:** A summary of the journal databases searched, the search terms used for each database, and the number of journals produced from each search

Database	Search terms	#Found
Web of Science	TI = ((neuro*) AND (marketing* OR consumer)) T2 = ((EEG* OR MRI* OR fMRI* OR pupil dilation* OR galvanic skin response OR eye-tracking OR eye-tracking) AND (marketing* OR consumer))	483
Science Direct	(neuro) AND (marketing OR consumer) (EEG OR MRI or fMRI OR pupil dilation OR galvanic skin response OR eye-tracking OR eye tracking) AND (marketing OR consumer)	349
Pubmed	(Neuromarketing) OR (Consumer neuroscience)	143
Wiley Online Library	Neuromarketing Consumer Neuroscience	129
Taylor & Francis	T1 = neuro AND (marketing OR consumer) T2 = (EEG OR MRI OR fMRI OR pupil dilation OR galvanic skin response OR eye-tracking OR eye-tracking) AND (marketing OR consumer)	124
ProQuest	neuromarketing OR "consumer neuroscience"	939
Total:		2167

Twenty journals were searched to find any articles that were not found in the database search. These journals were selected due to their focus on consumer behaviour, marketing psychology, and neuroscience. Individual journals were searched using the keyword 'neuromarketing'. Table 2 presents the journals searched and the number of articles generated for each.

**Table 2:** A table summarising the journals search for the present systematic review and the number of articles produced by each search

Journals	#Found
AJOB Neuroscience	9
Cognitive, Affective and Behavioural Neuroscience	0
European Journal of Marketing	11
European Journal of Neuroscience	0
Harvard Review of Psychiatry	1
International Journal of Consumer Studies	2
International Journal of Neuroscience	0
International Journal of Research in Marketing	3
International Journal of Technology Marketing	2
International Journal of Psychology	1
International Marketing Review	0
Journal of Computational Neuroscience	0
Journal of Consumer Affairs	1
Journal of Consumer Behaviour	14
Journal of Consumer Marketing	14
Journal of Consumer Psychology	5
Journal of Consumer Research	2
Journal of International Marketing	0
Journal of Marketing	0
Journal of Marketing Management	4
Journal of Marketing Research	5
Psychology and Marketing	6
Total	80

### Inclusion/exclusion criteria

The inclusion criteria defined for the systematic review were as follows:

- Primary study, published in a peer-reviewed journal.
- Studies that explored marketing using brain or physiological mechanisms, underlying theories of marketing, consumer behaviour, psychology and neurology.
- Research papers that used neuroimaging techniques such as EEG, fMRI, and positron emission tomography (PET) or physiological measures such as ET and GSR, to further understanding and application of marketing methods.
- Research papers using exclusively human, non-clinical populations as participants.

Studies that explored new developments in neuromarketing or summarised current research were classed as review papers and not included in the review. However, review papers were screened for citations, and any relevant citations which were not included in the original search were added to the systematic review.

The exclusion criteria for the systematic review were defined as:

- Any other literature review on Neuromarketing was excluded from the review.
- Articles that were not published in peer-reviewed academic journal articles were excluded (e.g., book chapters, post-graduate theses, conference abstracts).
- Articles written/published in any language other than English were excluded from the review.

### Screening process

Overall, 2247 articles were found in the literature review. The titles and abstracts of these articles were individually analysed by multiple researchers, who screened the papers according to the pre-defined inclusion and exclusion criteria.

#### Process

1. Read all titles, exclude any duplicates and those that are clearly not relevant according to exclusion criteria.
2. Read abstracts of those remaining, exclude any that are not relevant according to exclusion criteria.
3. The remaining papers qualify for full-text screening.
4. Of those that qualify, search through their reference list and all published articles which cite them.
5. Compile this list and perform title and abstract screening (stages 1–3) again.
6. Repeat as many times as necessary until the compiled list from stage 5 yields no papers that qualify for full-text screening.

Following the first stage of article accumulation, articles were exhaustively screened by the authors through title and abstract reviews and were categorised according to whether they matched the inclusion or exclusion criteria. 2512 papers were excluded from the literature review, while 777 were included. Of the journals excluded from the review, 516 were duplicate articles, 268 were neuromarketing review papers, and 930 were removed according to the pre-defined exclusion criteria. 21 additional papers were extracted from the review papers, and these were added to the included from the initial literature search, meaning 798 papers were included in the literature review. A further 8 papers were included in the literature review due to a second search being conducted at a later date using the same search terms.

To aid the analysis of the collected data, papers were separated into four discrete categories based on the measures used. The first category included papers that used EEG measures to investigate consumer or marketing-related behaviour, in which 213 papers were identified. The second category included papers that used neuroimaging measures based on blood oxidation levels such as fMRI or FINRS, in which 158 papers were found. The third category described studies that used physiological measures such as ET or GSR, in which 410 studies were found.

All papers that used EEG or MEG measures were included in the current subsection of the systematic review. However, some of the papers included in the EEG subsection also used physiological methods such as ET or the GSR, and facial action coding and were therefore included in both the EEG and physiological subsections of the systematic review. EEG papers were categorised according to whether they primarily used ERP or TF analysis methods. Table 3 summarises the papers included in the EEG subset of the systematic review, along with the relevant measures used (ERP, TF, Mixed-Methods, Machine-learning).

**Table 3:** A summary of papers included in the current subset of the systematic review, categorised according to the measures used

Categories	Studies included
ERP	Astolfi, Laura et al. [12]; Bastiaansen et al. [25], Bosshard et al. [37], Cai et al. [41], Camarrone and Van Hulle [42], Chen et al. [49], Daugherty et al. [62], Deitz et al. [63], Fan and Zhang [76], Fan et al. [75], Fu et al. [79], Fudali-Czyz et al. [80], Gajewski et al. [81], García-Madariaga et al. [83]; Gkaintatzis et al. [86],Goto et al. [91], Goto Nobuhiko et al. [92, 97, 100, 105, 106, 107, 111, 113], Jia et al. [124], Jin et al. [125], Jin et al. [126], Jing et al. [128], Jones et al. [129], Junghoefer et al. [131], Khushaba et al. [137], Kuan et al. [148], Kumagai et al. [150], Kytö et al. [154], Lee Hyun–Woo [160]; Li and Bu [165], Liao et al. [166], Luan et al. [170, 174, 176, 181, 174, 176, 181], Ma et al. [175], Ma et al. [179], Ma et al. [180], Ma et al. [177], Ma et al. [174, 176, 178, 181], Nittono and Watari [198], Ozkara and Bagozzi [205], Pileliene and Grigaliunaite [211], Pozharliev et al. [215], Roberts et al. [223], Royo et al. [231], Schaefer et al. [236], Shang et al. [248], Shang et al. [246], Shang et al. [247], Shen et al. [249], Soria Morillo et al. [255], Telpaz et al. [258], Thomas et al. [259], Treleaven-Hassard et al. [261], Tyson-Carr et al. [262], Uva et al. [264 , 279, 282, 284, 285], Wang and Han [279, 281, 282, 284, 285], Wang et al. [286, 297, 297, 299, 306, 310, 311, 312], Zubair et al. [315]
Time–frequency	Adrián et al. [1], Aldayel et al. [2], Aldayel et al. [3], Aprilianty and Purwanegara [9], Astolfi et al. [12, 13, 14], Ausin-Azofra et al. [15], Avinash et al. [16], Babiloni et al. [17], Balconi et al. [19], Balconi et al. [20], Baldo et al. [22], Baldo et al. [21], Barnett and Cerf [24, 27, 28], Berezka and Sheresheva [29], Bhushan et al. [31], Bigne et al. [33], Boksem and Smidts [34], Boshoff [35], Boshoff [36], Braeutigam et al. [38], Brown et al. [39], Caratu et al. [43], Cartocci et al. [45], Cartocci et al. [44], Cartocci et al. [46], Chen, et al. [50], Cherubino et al. [52], Christoforou et al. [53], Clark et al. [54], Constantinescu et al. [56], Correa et al. [58], Daugherty et al. [61], Di Gruttola et al. [65], Diao et al. [66], Dimpfel [68], Dulabh et al. [70], Eijlers et al. [71], Fallani et al. [74], Fischer et al. [77], Garcia-Madariaga et al. [82], Garcia-Madariaga et al. [83], García-Madariaga et al. [83], Garczarek-Bak and Disterheft [84], Gauba et al. [85], Golnar-Nik et al. [87], González-Morales [88], Goode and Iwasa-Madge [89], Gordon et al. [90], Gountas et al. [94], Guo et al. [99], Guo et al. [98], Guo and Elgendi [101], Hakim et al. [102], Hakim et al. [103, 105, 106], Harris et al. [109], Herrando et al. [110], Horska et al. [115], Horská and Berčík [116], Hsu and Chen [117, 118, 119], Hungenberg et al. [120], Janić et al. [122], Jeong and Kim [123], Kacaniova and Vargova [132], Khushaba et al. [138], Khushaba et al. [139], Khushaba et al. [137], Kim et al. [141], Kong et al. [145]; Kong et al. [146]; Kuan et al. [148], Kumar et al. [151], Laurence and Gerhold [157], Leanza [158], Lee et al. [159], Lee Eun-ju et al. [161]; Lee and Eun-Ju [162]; Lucchiari and Pravettoni [171],Mahamad et al. [182], Mengual-Recuerda et al. [189, 191, 192, 191, 192], Morey [194], Moya et al. [195], Nomura and Mitsukura [199], Ohme and Matukin [201], Ohme et al. [203], Ohme et al. [204], Ohme et al. [202], Pennanen et al. [207], Ramsøy et al. [217], Raiesdana and Mousakhani [216], Ramsøy et al. [218], Ravaja et al. [219], Rosenbaum et al. [226–228], Rothschild and Hyun [229]; Russo et al. [232], Alan Dos Santos and Moreno [5]; Schoen et al. [239], Senecal et al. [243], Shaari et al. [245], Shestyuk et al. [237], Slanzi et al. [251], Smith and Gevins [253], Soria Morillo et al. [254], Soria Morillo et al. [255], Touchette and Lee [260], Varan et al. [265], Vecchiato, et al. [266, 267, 268, 269, 270–272,273, 274], Vecchiato [266]; Vecchiato [267, 269, 273, 274, 278] Wajid et al. [277], Walsh et al. [278], Wang et al. [283], Wei et al. [291], Yadava et al. [294], Yang [295, 296, 298], Yazid et al. [300], Yen and Chiang [301], Yılmaz et al. [303], Young [305], Zhu et al. [314]
Mixed measures	Adrián et al. [1], Ausin-Azofra et al. [15], Baldo et al. [22], Barnett and Cerf [24], Bigne et al. [32], Berčík et al. (2021); Boshoff [35], Boshoff [36], Cartocci et al. [44], Cartocci et al. [46], Chen et al. [50], Cherubino et al. [52], Christoforou et al. [53], Clark et al. [54], Correa et al. [58], Dimpfel [68], Garcia-Madariaga et al. [82], Garcia-Madariaga et al. [83], Guo et al. [99], Herrando et al. [110], Horska et al. [115], Janić et al. [122], Mengual-Recuerda et al. [189, 191, 192], Moya et al., [195], Ohme et al. [203], Pozharliev et al. (2022); Russo et al. [232, 266, 267, 269, 270–274], Walsh et al. [278], Zhu et al. [314]
Machine-learning	Adrián et al. [1], Aldayel et al. [2], Aldayel et al. [3], Alimardani and Kaba [4], Al-Nafjan [6], Ausin-Azofra et al. [15], Bandara et al. [23], Barnett and Cerf [24], Bhushan et al. [31], Bigne et al. [32], Boshoff [35], Boshoff [36], Cartocci et al. [44], Cartocci et al. [46], Chen et al. [50], Cherubino et al. [52], Christoforou et al. [53], Clark et al. [54], Correa et al. [58], Dimpfel [68], Garcia-Madariaga et al. [82], Garcia-Madariaga et al. [83], Gauba et al. [85], Golnar-Nik et al. [87], Guo et al. [99], Guo et al. [98], Guo and Elgendi [101], Guixeres et al. [96], Hakim et al. [102], Hakim et al. [103], Khushaba et al. [139], Kumar et al. [151], Ma and Zhuang [173]; Mashrur et al. [185], Mashrur et al. [186], Mengual-Recuerda et al. [189, 191, 192], Moya et al. [195], Ohme et al. [203], Pandey et al. [206], Phutela et al. [210], Roberts et al. [223], Russo et al. [232], Shestyuk et al. [237], Slanzi et al. [251], Soria Morillo et al. [254], Soria Morillo et al. [255], Tyson-Carr et al. [262], Ullah et al. [263, 266, 267, 269, 270–274], Walsh et al. [278], Wang et al. [287], Wei et al. [291], Yadava et al. [294], Yılmaz et al. [303], Zamani and Naieni [307]; Zeng et al. [308], Zheng et al. [313], Zhu et al. [314]

#### Secondary literature search

In order to identify neuromarketing literature which used machine-learning algorithms to predict consumer behaviour based on EEG signals, a secondary literature search was conducted. Literature searches using the six journal databases used in the primary literature search were conducted using the search terms “consumer neuroscience”, “neuromarketing”, and “machine learning”. From this literature search, an additional eighteen studies were identified which used machine learning to predict consumer preference or purchase intention using EEG signals [4, 6, 23, 24, 87, 96, 185, 185, 186, 186, 206, 210, 237, 263, 287, 291, 294, 307, 308, 313]. These additional studies have been included in the systematic review section.

### Data synthesis

In the data synthesis step, an aggregative approach was used to summarise the conclusions of the literature. Such an approach depends on the subjective interpretation of the researchers concerning the reviewed articles and, considering this, a certain degree of subjective latitude should be given to enable researchers to evaluate and compare distinct studies, with the purpose of extracting shared meanings and abstracting the approaches that do not concern the purposes stated for the review [73].

The overall objective of the present subset is to provide a mapping of the consistency and direction of significant EEG effects found in neuromarketing research, identifying specific components showing strong effects or consistencies. The results were be analysed using pattern correspondence [64].

## Systematic review

### EEG introduction

Cortical oscillatory activity can be analysed using three critical forms of information extracted from the waveform; amplitude, phase, and frequency [143]. Amplitude reflects the size of a peak in terms of its volts, while frequency measures how many oscillations occur per second (Hz), and phase represents the relative position of the wave in time. Using these forms of information, EEG data are typically analysed using one of two approaches; time–frequency analysis (TF) or event-related potential analysis (ERP).

### Time–frequency analysis

EEG TF measures can expand on traditional marketing measures such as self-reports and behavioural willingness to pay by showing the underlying cognitive processes behind participant decision-making or the effect of specific changes to features of products or advertisements. EEG TF measures can further expand on physiological measures of arousal such as GSR by unveiling the underlying cognitive and emotional processes behind product and advertisement evaluation.

During time–frequency analysis, changes in the power of cortical oscillations are analysed according to pre-defined frequency bands, usually time-locked and averaged to a particular class of events [183]. Lower frequency bands generally exhibit larger amplitudes than higher frequency bands and usually reflect more extensive patterns of cortical activation [197]. Power changes can be analysed primarily according to a baseline condition (as is done in relative-band power or event-related desynchronisation analysis) across the scalp or relative to another region of the scalp (as is done in studies using measures of frontal asymmetry).

#### Frontal asymmetry

Consumer neuroscience is often focused on separating positive and negative responses to sales and marketing stimuli [63] to modify or predict consumer choices through behavioural, physiological, or neural measures [267, 269, 273, 274]. The relative difference in power between the left and right prefrontal cortex, especially in the alpha frequency band, has emerged as a critical measure separating positive from negative responses [252]. This neural marker is generally interpreted as reflecting the motivational direction and preference towards a stimulus and often occurs just before the formation of behavioural intentions [108, 112, 275]. Frontal asymmetry is posited to reflect an approach response to stimuli when indicating an increase in cortical activity to the left side and an avoidance response when indicating an increase in cortical activity to the right side [108, 112]. For this reason, frontal asymmetry is thought to be a more nuanced measure of preference than physiological measures such as GSR, which can only identify valence magnitude, not direction [108, 112].

The relative degree of alpha frontal asymmetry is calculated using the following formula:

$$\frac{Left \,alpha \,power - right \,alpha \,power}{left \,alpha \,power + right\, alpha\, power}*100.$$

Frontal asymmetry was commonly found during positive elements of viewed advertisements [15, 52, 108, 112, 157, 182, 203, 204, 227, 277], as well as proving useful in the prediction of advertisement preference and success [46, 51, 54, 157, 182, 203, 204, 266, 266, 270, 270, 271, 271, 272, 272, 275, 277]. Frontal asymmetry has also been shown to differentiate between emotional responses to advertisements by gender [267, 269, 273, 274] and age [267, 269, 273, 274] and shows approach/avoidance responses to marketing-related stimuli such as food [39, 207, 232, 278], music [16] and sales/persuasion messaging [58, 65].

Frontal asymmetry has also been found to be predictive self-reported preference [9, 110, 118, 119, 122, 148, 191, 192, 260], and emerged as the only EEG TF measure that is consistently associated with behavioural measures of willingness to pay (WTP) [50, 138, 148, 217–219, 239]. This suggests that frontal asymmetry may reflect actional/motivational responses to brands/products while evaluative ratings and recall may be better investigated using other TF measures such as relative-band power changes [26, 87, 99, 115, 117, 138, 145, 226, 237]. For example, Ramsøy et al. [217] and Ramsøy et al. [218] showed using a principal component analysis that prefrontal asymmetry best accounted for variance in WTP, while other TF measures best accounted for self-reported preference.

While frontal asymmetry is most commonly associated with alpha-band activity [108, 112, 275], effects were also reported in the theta and beta bands in the reviewed literature. Frontal asymmetry is most widely reported in the alpha band and appears to reflect WTP and advertisement effectiveness, therefore likely reflecting the motivational approach response most commonly associated with frontal asymmetry [44–46, 50, 65, 148, 157, 182, 191, 192, 203, 204, 218, 219, 227, 237, 239, 267, 267, 269, 269, 273, 273, 274, 274, 275, 277, 278]. Frontal asymmetry reported in the theta-band mostly related to self-reported preference [16, 138, 266, 270–272, 275], and advertisement memorability [44, 46, 182, 266, 266, 270, 270, 271, 271, 272, 272]. Further, while two experiments reported significant changes in beta-band frontal asymmetry [9, 182, 217], the cognitive processes reflected by this response seem much less certain. Therefore, it is recommended that frontal asymmetry measures are primarily employed in the alpha band and are best used in neuromarketing when assessing approach/avoidance responses to advertisements and willingness to pay towards products.

#### Relative-band power changes

While frontal asymmetry is considered to be the best measure of motivational valence, relative-band power changes in specific frequency bands can be used to measure other cognitive and emotional responses to marketing stimuli. For example, a reduction in power in the alpha band (8–12 Hz) over occipital areas is generally considered to reflect visual processing. Occipital alpha-band power was found to be modulated by video but not print advertisements in early experiments [62], and has been used in more recent experiments to measure the visual processing of advertisement features [83, 147, 228, 229, 253, 300].

Frontal alpha-band suppressions are linked to information processing, attentional orienting, decision-making and emotional regulation [55, 143, 190, 309], and were shown in the reviewed literature to relate to advertisement recall and effectiveness [68, 71, 74, 90, 109, 145, 267, 269, 273, 274] (Pozharliev 2022), TV viewership [237] and self-reported preference [26, 82, 83, 118, 119] as well as responding to preferred brands and pro-social products [146, 161, 244]. Power changes in the alpha band over frontal regions of the scalp can therefore be considered to be a helpful neuromarketing tool, especially when considering the saliency of advertisement and product features.

Theta rhythms describe slower and larger oscillatory frequencies within the 4–7 Hz range [235]. An increase in midline theta power is reliably associated with long-term memory encoding [95, 142, 234] and an increase in sustained effortful engagement [47, 121, 169]. The papers reviewed showed that theta-power was commonly associated with memorable elements of advertisements [68, 70, 74, 132, 145, 266, 266, 267, 267, 269, 269, 270, 270, 271, 271, 272, 272, 273, 273, 274, 274], recognised brands [68, 70, 74, 132, 145, 146, 171, 191, 192, 195, 245, 266, 266, 267, 267, 269, 269, 270, 270, 271, 271, 272, 272, 273, 273, 274, 274] and out-of- and within-sample success [44, 46, 68, 70, 74, 83, 90, 109, 117, 132, 145, 146, 158, 171, 191, 192, 195, 237, 245, 258, 266, 266, 267, 267, 269, 269, 270, 270, 271, 271, 272, 272, 273, 273, 274, 274]. However, preference associations were less common [19, 20, 66, 118, 119, 160–162, 216, 244, 295]. Increases in theta-band power can be considered a valuable tool in advertising research, highlighting the memorability and out-of-sample effectiveness of tested advertisements, but may be less useful in investigating buying behaviour or preference.

Faster cortical oscillatory activity found in the beta (16–24 Hz) and gamma (30–45 Hz) ranges are less clearly interpreted in neuromarketing research. Beta-band power is traditionally associated with movement preparation and intention formation when suppressed over sensorimotor regions [72, 208, 209], as well as inhibition when increased over right frontal areas [40, 276], while gamma-band over prefrontal areas is associated with visual attention [233], working memory [93, 230], and language abilities [93]. However, in the literature, beta and gamma band changes were modulated by a range of stimuli, including advertisement memorability [13, 14, 17, 74, 194], preference [53, 89, 115, 158, 171, 296], emotional valence [89, 296, 298], and changes in the shopping environment [27, 28, 116]. Therefore, relative-band power changes in the beta and gamma bands should be treated with caution when used in neuromarketing research. Further investigation is required to identify their exact role in buying and advertising behaviour.

Other EEG TF measures have been used in neuromarketing research, such as cross-brain correlations across two participants predicting advertisement preference and recall [24], or global field power and peak density function [13, 14, 94, 105, 106, 266, 266, 266, 267, 269, 270, 270, 270, 271, 271, 271, 272, 272, 272–274], and partial directed coherence [74]. Additionally, pre-defined emotion toolboxes have been used to gauge emotional responses to marketing stimuli based on EEG TF responses [21, 63, 115, 199, 231]. However, due to the limited research done, it is difficult to judge the consistency of these measures, so further research is required.

#### Mixed measures experiments

A subsection of the studies reviewed used a mix of EEG time–frequency measures and physiological measures; including measures of arousal, such as the GSR heart-rate variability (HRV) and pupil dilation (PD); ET measures of attentional orienting; and facial-expression analyses. The use of a mixed-measures design allows for the comparison of EEG TF measures with physiological measures, identifying the strengths and weaknesses of each and determining which measures should be used in which contexts [56].

Several studies have shown that measures of arousal and EEG TF responses are modulated by advertisement preference and memorability [1, 44, 46, 58, 83, 203, 266, 266, 267, 267, 269, 269, 270, 270, 271, 271, 272, 272, 273, 273, 274, 274], as well as differences in product features [43, 50, 278]. However, physiological measures of arousal appear to be unable to differentiate between different emotional and cognitive responses, while EEG TF measures can [1, 22, 24, 50, 52, 83, 110, 189, 191, 192, 195, 232, 266, 273]. For example, [50] Chen [49] showed that HRV measures of arousal could only differentiate between the intensity of mouthwash flavours, while FAA distinguished between flavours and was predictive of self-reported preference and purchase intention. It, therefore, appears that physiological measures of arousal are less useful when combined with EEG TF measures due to their lack of sensitivity.

ET, or the tracking of eye movements, is a measure that can easily be combined with EEG measures while participants view an advertisement or product [53, 54, 68, 82, 83, 99, 191, 192, 195, 267, 269, 273, 274]. The advantage of this measure over EEG TF measures is that it can be used to identify product or advertisement features that draw attention within a visual field [15, 32, 53, 68, 82, 83, 99, 189, 267, 269, 273, 274, 287] (Pozharliev et al. 2022). Further, Zhu et al. [314] found that, while EEG can be used to build more accurate machine learning models of customer preference than ET, the inclusion of ET data does improve the predictive accuracy of the model when compared to models using EEG data alone. ET, therefore, provides a useful and complementary measure to EEG TF measures. Some papers found significant attentional ET effects, but no significant EEG differences [32, 82]. ET should therefore be considered for use with EEG measures.

Only eight studies identified have combined facial expression analysis or EMG with EEG TF analyses [15, 35, 36, 54, 115, 203, 278] (Berčík et al. 2021). However, this measure provided a useful complement to EEG TF measures, as facial expressions and micro-expressions can separate different emotional responses such as happiness and disgust. Although the combination of EEG TF and facial-expression analysis is currently not well employed in neuromarketing research, further exploration should be pursued.

#### Machine-learning prediction

In more recent years, neuromarketing researchers have begun to use machine-learning classification of EEG TF measures to improve the prediction of ‘like/dislike’ or pleasantness ratings. Early studies primarily used multivariate analysis methods, such as logistic regressions, to predict preference ratings [24, 85, 101, 139, 237, 303]. The subsequent use of machine-learning algorithms has been shown to improve the predictive accuracy of a model above the use of traditional logistic regressions [31, 85, 87, 96, 102, 103, 151, 185, 185, 186, 186, 251, 255, 287, 291, 294, 313]. Most studies reviewed employed the use of multiple machine-learning algorithms, allowing for the direct comparison of these methods [2–4, 6, 23, 87, 98, 102, 103, 206, 210, 251, 254, 263, 287, 294, 307, 308] (see Table 4).

**Table 4:** A systematic table summarising the machine-learning studies included in the present review, including the features, labels, and algorithms used, as well as the accuracy rates achieved

Citation	Features	Labels	ML algorithms	Accuracy rates	DOI
Aldayel et al. [2]	DEAP dataset, power spectral density and asymmetry	Preference ratings	DNN, KNN, SVM, RF	0.94, 0.88, 0.62, 0.92	https://doi.org/10.3390/app10041525
Aldayel et al. [3]	DEAP dataset, power spectral density	Preference ratings	KNN, SVM, RF, DNN	0.73, 0.81, 0.87, 0.83	https://doi.org/10.3389/fnhum.2020.604639
Al-Nafjan [6]	PCA, mRMR, RFE, ReliefF feature selection algorithms	Like/Dislike self-report responses to Commercial products	DNN, SVM, KNN, LDA, RF	0.93, 0.81, 0.78, 0.70, 0.82	https://doi.org/10.7717/peerj-cs.944
Alimardani and Kaba [4]	Raw EEG signal	Product Choices and movie ratings	CNN, Ensemble Model	0.51–0.75, 0.51–0.64	https://doi.org/10.1145/3460881.3460930
Bandara et al. [23]	PCA feature selection from power spectral density	Movie trailer preference	RF, SVC	91.97, 91.70	https://doi.org/10.1109/ICIIS53135.2021.9660742
Barnett and Cerf [24]	EEG cross-brain correlation	Product willingness to pay	Linear Regression	0.66	https://doi.org/10.1093/jcr/ucw083
Bhushan et al. [31]	TF synchronisation	Binary preference	ANN	74.3	https://doi.org/10.1371/journal.pone.0043351
Gauba et al. [85]	EEG TF effects	Video advertisement preference	RF, DT, Linear regression	0.68, 0.33, 0.041	https://doi.org/10.1016/j.neunet.2017.01.013
Adrián et al. [1]	HR, GSR and EEG TF effects	Video advertisement Valence score	Logistic Regression, SVM, RF	0.66, 0.67, 0.89	https://doi.org/10.3389/fncom.2016.00074
Golnar-Nik et al. [87]	EEG power	Like/dislike ratings	SVM, LDA	0.87, 0.90	https://doi.org/10.1016/j.physbeh.2019.04.025
Guo and Elgendi [101]	TF effects, prepurchase ratings	E-commerce purchase behaviour	Recommender system	N/A	https://doi.org/10.12720/joams.1.1.61–65
Guo et al. [98]	ET, and TF power	Aesthetic preference	SVM, KNN, RF, XGBoost	0.54, 0.61, 0.59. 0.56	https://doi.org/10.1016/j.ergon.2019.02.006
Guixeres et al. [96]	EEG, HRV, ET	Youtube advertisement like/dislike and recall scores	ANN	0.83	https://doi.org/10.3389/fpsyg.2017.01808
Hakim et al. [102]	Frontal band power, hemispheric asymmetry, inter-subject correlations	Product preference	SVM, Logistic regression, DT, KNN,	0.69, 0.67, 0.63, 0.60	https://doi.org/10.1101/317073
Hakim et al. [103]	Frontal band power, hemispheric asymmetry, inter-subject correlations	Product Preference	SVM, Logisitic Regression, KNN, TREE	0.69, 0.67, 0.63, 0.60	https://doi.org/10.1016/j.ijresmar.2020.10.005
Khushaba et al. [139]	Eye-tracking fixation and time–frequency effects	Product preference	Logistic Regression	N/A	https://doi.org/10.1016/j.eswa.2012.12.095
Kumar et al. [151]	Raw EEG signal	Product valence scores	RF	0.74	https://doi.org/10.1016/j.inffus.2018.11.001
Pandey et al. [206]	Discrete Wavelet Transform power and entropy for 5 frequency bands	Movie trailer Likert ratings (rating, familiarity, purchase intent, willingness to spend)	KNN, RF, MP	0.72, 0.71, 0.67	https://arxiv.org/abs/2007.10756
Phutela et al. [210]	Wavelet coefficient, power spectral density, and Hjorth parameters	Advertisement and product preference	NB, SVM, KNN, DT, DL	0.66, 0.66, 0.55, 0.54, 0.55	https://arxiv.org/abs/2206.07484
Ma et al. [178]	ERP	Brand extension acceptance rates	T-SNE algorithm	0.87	https://doi.org/10.3389/fnhum.2021.610890
Mashrur et al. [185]	Frequency, time, and time–frequency metrics over frontal electrodes	Purchase intention and affective attitude towards advertisements	SVM	0.84	https://doi.org/10.3389/fnhum.2022.861270
Mashrur et al. [186]	Frequency, time, and time–frequency metrics over frontal electrodes	Affective attitude towards E-commerce products	SVM	0.94	https://doi.org/10.1016/j.physbeh.2022.113847
Roberts et al. [223]	ERP and eye-movements	Product willingness to pay	Independent component analysis	N/A	https://doi.org/10.3389/fnins.2018.00910
Shestyuk et al. [237]	Frontal asymmetry (alpha/beta), fronto-central power (alpha/theta + theta/gamma)	TV viewership and twitter activity	Linear regression, multiple regression	0.63, 0.72	https://doi.org/10.1371/journal.pone.0214507
Soria Morillo et al. [254]	Frequency band power	Advertisement like/dislike ratings	ANN	0.75	https://doi.org/10.1186/s12938-016–0181-2
Soria Morillo et al. [255]	Frequency band power	Advertisement like/dislike ratings	ANN	0.82	https://doi.org/10.1007/978–3-319–16,480-9_68
Slanzi et al. [251]	Gaze position, pupil dilation, and EEG TF	Website click choice	SVC, logistic regression, neural network	0.69, 0.71, 0.62	https://doi.org/10.1016/j.inffus.2016.09.003
Tyson-Carr et al. [262]	ERP and eye-movements	Product willingness to pay	Independent component analysis	N/A	https://doi.org/10.1016/j.neuroimage.2019.116213
Ullah et al. [263]	Wavelet transformed EEG data	E-commerce product like/dislike ratings	SVM, DT, KNN, ANN	0.80, 0.68, 0.76, 0.81	https://doi.org/10.14569/IJACSA.2022.0130137
Wang et al. [287]	EEG and ET data	Self-reported preference	PCA, Random Lasso, SVM	0.80, 0.75,0.92	https://doi.org/10.1016/j.aei.2020.101095
Wei et al., [291]	EEF TF data	Advertisement effectiveness	SVM	0.75	https://doi.org/10.3389/fnins.2018.00076
Yadava et al. [294]	EEG TF effects	Like/dislike ratings of e-commerce products	HMM	0.68	https://doi.org/10.1007/s11042-017–4580-6
Yılmaz et al. [303]	Power spectral density	Product like/dislike ratings	Logistic regression	N/A	https://doi.org/10.1016/j.cmpb.2013.11.010
Zamani and Naieni [307]	Band power over five brain lobes	Like/dislike ratings of E-commerce products	SVM, ANN, RF	0.87, 0.70, 0.81	https://doi.org/10.18502/fbt.v7i3.4621
Zeng et al. [308]	Power spectral density, hemispheric asymmetry, differential entropy, Hjorth parameters	Sport shoes like/dislike ratings	KNN, SVM	0.94, 0.8	https://doi.org/10.3389/fnhum.2021.793952
Zheng et al. [313]	PSD	Emotion classification	DNN	0.85	https://doi.org/10.1109/TCYB.2018.2797176
Zhu et al. [314]	EEG and ET mixed measures	Self-reported preference	SVM, RF, CNN	70.26, 72.15, 96.4	https://doi.org/10.1016/j.aei.2022.101601

Across the reviewed literature, the most commonly used classification methods were DNN [2, 3, 6, 313], KNN [2, 3, 6, 98, 102, 103, 206, 210, 263, 308], SVM [1–3, 6, 87, 98, 102, 103, 185, 185, 186, 186, 210, 263, 291, 307, 308, 314], RF [1–3, 6, 23, 85, 98, 151, 206, 307, 308, 314], and regressions [1, 24, 85, 102, 103, 138, 237, 251].

The highest predictive accuracy reported was found by experiments using DNN algorithms, which achieved a binary classification accuracy of 85–94% (M = 0.89, SD = 0.06), followed by RF algorithms (M = 0.80, SD = 0.11), then SVM algorithms (M = 0.77, SD = 0.11), and KNN algorithms (M = 0.72, SD = 0.13). The worst classification accuracy was reported by studies using regression methods, which were only able to correctly classify consumer preference around 60% of the time (M = 0.59, SD = 0.24).

Machine-learning prediction appears to be a fruitful avenue of research within neuromarketing and can achieve very high predictive accuracy, potentially overcoming the reliability problems identified in earlier portions of the systematic review. However, there are several additional methodological considerations that researchers must take into account when using machine-learning prediction. Crucially, machine-learning algorithms may require a larger number of trials and greater computational power than traditional regression models, due to the complex calculations required [136]. Machine learning models are also vulnerable to ‘overfitting’, where models achieve high accuracy rates for training data, but perform poorly when predicting out-of-sample values [67, 225, 302]. Current neuromarketing research using machine-learning prediction also seems relatively limited, primarily focusing on product preference, so this method should be expanded in the future.

### Event-related potential measures

ERPs reflect averaged transient effects to specific stimuli or a specific class of stimuli [172, 183, 238]. ERPs, therefore, measure phasic responses to advertising stimuli, occurring within hundreds of milliseconds following stimulus onset [172, 238], and thus capitalise on the high temporal resolution of EEG recordings. It is generally considered that reactions that occur within the first 300 ms of decision-making are unconscious, while those occurring after 300 ms are related to conscious inclinations [165, 193]. The consensus regarding whether the P300 and N400 components reflect conscious or unconscious reactions remains contentious, and these components may reflect a critical phase in the transition from unconscious to conscious mental processes [57, 156, 250].

#### N400/N200 ERP component

The N200 and N400 components are most commonly associated with conflict and unfamiliarity, especially brand extension and recognition [48, 152, 240, 292]. The N200 is a negative potential peaking between 200 and 350 ms after stimulus onset, with an amplitude that is negatively related to familiarity and is generally considered to represent fast and unconscious conflict processing [48, 152, 240, 292]. The N400, a negative potential peaking around 400 ms following stimulus onset, is commonly related to violations of grammatical rules and unexpected stimuli. N400 amplitude is thought to reflect the corresponding conscious processing of conflicting information [48, 152, 240, 292]. These components are most commonly used in neuromarketing research to measure consumer familiarity with brands and products and the conflict between price and expected value. In the reviewed literature, fourteen studies using brand-extension paradigms reported modulations in N200/N400 amplitude [42, 76, 80, 125, 175, 177, 179, 180, 246, 248, 286, 295, 299, 312], nine studies reporting N200/N400 modulations used conflict tasks such as oddball tasks [79, 81, 97, 100, 107, 126, 129, 205, 259, 311], and nine used other tasks, such as auction tasks or advertising stimuli [91, 92, 105, 106, 124, 128, 137, 279, 279, 282, 282, 284, 284, 285, 285, 310].

In the literature, significant N200 and N400 amplitude differences were most commonly found in experiments utilising brand extension paradigms. Brand extension tasks are used to investigate how generic product types (e.g., coffee) are associated with brand names or logos (e.g., Starbucks). Twelve out of the 15 studies which investigated brand extension in the reviewed literature found significant effects in N200 or N400 amplitude [76, 80, 125, 177, 179, 180, 246, 248, 286, 297, 299, 312], while one used machine-learning and therefore did not investigate modulations in ERP amplitude directly [178]. Within the 11 studies that reported significant N200/N400 effects, nine reported significant effects of N400 amplitude [42, 80, 125, 177, 180, 246, 286, 295, 299], two reported significant N200 effects [76, 179], while only two studies found significant effects in both N400 and N200 amplitude [80, 299]. Further, two studies reported non-significant N2 amplitude modulations [297, 299]. In contrast, six of the studies using brand extension experiments reported significant differences in P300 amplitude, and only two found significant LPP or LPC differences [76, 246]. N200 and N400 amplitudes were generally interpreted as reflecting conflict processing and were negatively related to brand extension acceptance rates [42, 76, 80, 177, 179, 180, 246, 286, 295].

N400 and N200 amplitudes have also been associated with perceptions of brands and products, especially when conflicted with other relevant features such as reviews or previous experiences. It was found by nine of the reviewed studies that N200/N400 amplitudes were significantly modulated by participant awareness of a brand/product [105–107, 175, 279, 282, 284, 285, 310, 311]. When elicited during oddness experiments, N200/N400 amplitudes were most commonly modulated by incongruence caused by negative framings or reviews during the viewing of a product or brand [49, 81, 97, 100, 126, 166, 205], as well as product preference [91, 92, 124, 137, 175, 279, 281, 282, 284, 285]. Three studies investigated the effect of price on N400 and N200 amplitudes [79, 129, 137] and found that amplitudes were modulated by violations in price expectations and price deception.

Overall, the N200 and N400 ERP components are typically used in neuromarketing research to identify brand familiarity, extension, and conflict caused by negative attitudes and price violations. Reported effects of preference on N400/N200 amplitudes were less consistent and should be treated with caution. Significant effects were more commonly found in N400 than N200 amplitudes, suggesting that response conflict occurs more consciously in consumers.

#### P300/P200 ERP component

The P2 and P300 ERP components are positive potentials occurring between 200 and 400 ms after cue onset. It is generally considered that P300 amplitude is positively related to the attentional resources allocated towards a stimulus [133, 140, 196]. The P2 is a similar positive potential, which peaks approximately 200 ms after stimulus presentation [114, 280, 293], and is considered to reflect the rapid automatic activity of attention [113, 114, 280, 293]. Based on the theoretical background of P300 and P200 modulations, it would be expected that their best use in neuromarketing would emerge in investigating how products and advertisements attract attention, and the best ways to draw attention.

In the present review, P300 and P2 amplitude were found to be especially effective in the investigation of advertisement [61, 62, 261, 279, 282, 284, 285, 289] and marketing [212, 215, 259, 264, 315] effectiveness, with significant amplitude modulations found in all reviewed studies. P300 and P200 amplitude were also significantly modulated by preference [25, 126, 137, 174, 174, 176, 176, 181, 181, 264, 281, 306], purchase intention [41, 92, 127, 129, 160, 162, 170, 174, 176, 181, 247], price [128, 223], and brand or product features [75, 81, 105–107, 311]. However, significant effects were not reported in all papers investigating these factors [37, 49, 79, 91, 92, 111, 131, 166, 205, 249, 258], so results may lack statistical power. Especially of note were the inconsistent effects found regarding preference on P200 amplitude, with some papers finding smaller amplitudes to preferred stimuli [107, 124, 126, 129, 170, 315], and others finding the reverse [25, 128, 174, 174, 176, 176, 181, 181, 306].

Overall, P300 and P2 amplitudes were revealed to be especially effective when investigating advertising effectiveness. However, modulations in these ERP components should be treated with caution when examining consumer preference as it may lack statistical power and may only be reflective of attention drawn due to stimulus salience rather than valence.

#### LPP component

The LPP is a positive component, usually found later than 400 ms following stimulus onset, and is generally considered to reflect conscious emotional processing. The LPP is sensitive to emotional stimuli, both positively and negatively valanced [78, 153, 241, 242], and has been proposed to represent emotional regulation processing or attention towards the emotional nature of stimuli. The LPP is commonly used in neuromarketing due to its relationship to conscious emotional evaluation, which is strongly related to purchase behaviour and brand perception [25, 37, 166, 174, 176, 181].

In the reviewed literature, the LPP was most commonly associated with preference or emotional evaluation towards products and brands [25, 37, 75, 79, 91, 92, 126, 128, 166, 174, 174, 174, 176, 176, 176, 181, 181, 181, 198, 215, 247, 249, 306, 310, 315], while only six papers investigating participant preference did not report significant modulations in LPP amplitude [62, 137, 154, 258, 261, 281]. However, the effect of emotional content on LPP amplitude appears to predominantly be reflective of valence strength rather than direction [128, 249, 315], meaning it may be unable to differentiate between positive and negative attitudes towards brands and products. The results reported by Goto et al. [92] further showed that when used to predict single-trial product preference, the LPP achieved the highest accuracy (70%) of all the ERP components investigated. However, it may be less sensitive under low trial numbers [107].

Taken together, the reviewed literature reveals the LPP as a key ERP component in neuromarketing research, as it directly reflects emotional evaluation of brands and products, rather than the correlated measures of attention and conflict. For this reason, the LPP may be a more appropriate measure in neuromarketing research for the investigation of preference. However, the LPP should be used with caution, as it may be unable to untangle emotional valence and may only be reflective of intensity.

#### Other ERP components

A limited number of studies identified in the literature review investigated further ERP components in relation to marketing stimuli, including the MMN [111], N1 [25, 97, 100, 205, 282, 289, 306], FRN [41, 236], LPC [129, 246, 279, 282, 284, 285], and PSW [92]. However, due to the limited number of studies investigating the effectiveness of these ERP components, it is difficult to make explicit judgments regarding their use and effectiveness.

#### ERP studies using machine learning/ICA

Three studies were identified that used independent component analysis or machine-learning on phase-locked EEG data rather than traditional ERP analysis. Tyson-Carr et al. [262] used an independent component analysis (ICA) to investigate ERP effects behind willingness-to-pay, finding that significant differences between EEG activity between the right and left parietal lobe at around 200 ms following stimulus onset were most predictive of willingness-to-pay. Similarly, Roberts et al. [223] differentiated between the phase-locked EEG activity found in response to high- and low-value items using ICA analysis. Ma and Zhuang [173] was the only study identified in the present systematic review which used machine-learning to investigate marketing-relevant stimuli based on phase-locked EEG activity. Using t-SNE machine learning, the researchers predicted brand-extension acceptance with an accuracy of 87.37%.

#### ERP conclusions

Taken together, the literature collected in the present review identified three ERP components that were most commonly used in neuromarketing research: N400, P300, and LPP. Modulations in N400 and P300 amplitude were best implemented when investigating specific neuromarketing effects such as conflict and attentional saliency. In contrast, LPP amplitude modulations appeared more suitable to measure preference and emotional evaluation, although it is only sensitive to magnitude, not valence. The use of alternate data analytic approaches such as machine learning and ICA is less common in ERP analysis than TF analysis. However, studies in this area seem promising. It is recommended that future ERP research in neuromarketing employs machine-learning and ICA analyses.

## Discussion

The results of the present systematic review revealed several key recommendations that can be made regarding the use of EEG measures in neuromarketing. First, key ERP and TF components were identified as the most consistent markers of preference and emotional evaluation, namely the FAA and LPP. Second, the importance of machine-learning analysis in future neuromarketing research was highlighted. Finally, it was shown that EEG measures are best used in conjunction with ET and facial expression analysis rather than GSR or PD.

The core finding of the present systematic review was the identification of FAA and LPP as key TF and ERP components in the investigation of consumer preference. Overall, FAA was judged to be the optimal measure of preference due to its ability to disentangle positive from negative responses, while the LPP only indexed response magnitude. Further components were identified that were useful in indexing customer attention (P300 amplitude, alpha-band power, theta-band power), memorability (theta-band power) and emotional conflict (N400 amplitude). These components should be considered in future neuromarketing research but not used as principal measures of consumer preference.

Traditional marketing models assume that consumer decisions are mostly rational, and therefore ignore the role of implicit emotional responses in consumer preference and buying decisions [7, 69]. Neuromarketing overcomes these limitations through the use of biometric and neuroimaging measures, which can detect implicit emotional responses traditionally ignored in marketing research [7, 69, 220]. The primary benefits of neuromarketing are therefore to improve the accuracy of models aiming to predict consumer preference and buying behaviour, and provide a greater understanding of the emotional impact of marketing stimuli on consumers [7, 69, 220]. Ultimately, neuromarketing research should be developed in ways that can be actively used to improve products or advertising campaigns. However, there was a large degree of inconsistency found in the reviewed literature regarding the significance and interpretation of different EEG effects in a marketing context, specifically when relating to consumer preference.

The present results, therefore contribute to the literature by demonstrating the most consistent EEG measures of consumer preference and willingness to pay, and these measures require greater focus in future research. Matching the theoretical literature, FAA appears to reflect approach/avoidance responses to stimuli [108, 275] and was the only component identified in the current review that could untangle positive from negative emotional reactions. In contrast, the LPP ERP component appears to reflect conscious emotional processing of marketing stimuli [78, 153, 241, 242] but cannot separate positive from negative responses. Future neuromarketing research should therefore focus on the use of the FAA and LPP when creating predictive models of consumer preference.

It would also be appropriate to use the other components identified as measures of factors that may indirectly relate to preference. For example, P300 amplitude and theta-band power appear to be effective measures when investigating advertisement effectiveness, reflecting attentional orienting [133, 140, 196] and memory encoding [95, 143], respectively. In contrast, the N400 ERP component was most effective in investigating brand extension acceptance rates. Future research investigating these components should therefore build their hypotheses based on these findings and not use them as undifferentiated measures of preference.

When used in a mixed-measures design, EEG data were found to be best combined with eye-tracking and facial-expression analysis, as these provide data types that EEG cannot reveal. ET measures are useful in demonstrating which areas of a visual field (e.g., advertisement) draw customer attention [32, 83]. Similarly, facial-expression analyses can reveal specific emotional responses to marketing stimuli such as joy or disgust [15, 54, 115, 203, 278]. In contrast, physiological measures of arousal such as GSR and PD provide less additional interpretive utility as they only reflect arousal intensity [1, 24, 50, 52, 83, 189, 191, 192, 195, 232], which can be indexed by EEG measures such as the FAA or P300.

It is our hope that the interpretive framework provided in the present review will aid in the analysis and interpretation of future neuromarketing research, and provide a neuromarketing-specific interpretation of EEG data, preventing post hoc analysis of future results. Highlighting the importance of a clear interpretive framework, significant inconsistencies were found across several sections of the systematic review, and future researchers should be aware of the issues of replication in neuromarketing research. For example, several studies found a positive relationship between preference and P2 amplitude [107, 124, 126, 129, 170, 315], while others found a negative relationship [25, 128, 174, 174, 176, 176, 181, 181, 306]. These inconsistencies reflect the larger replication crisis in psychology and may result from small effect sizes, cherry-picking of results, and the overuse of frequentist statistics.

Machine-learning algorithms are a potential solution to the replication problem, achieving high predictive accuracy in the reviewed literature, consistent across papers. The most effective machine-learning algorithms were DNNs, which reported accuracies as high as 94% in predicting consumer preference. The predictive accuracy of machine learning was further improved when conducted on EEG data combined with physiological measures. Therefore, the present results highlight the importance of machine-learning analyses in future neuromarketing research to improve the replicability and consistency of results.

While machine learning presents a promising avenue for neuromarketing research, care should be taken when designing machine learning models. First, due to the complex calculations made, machine learning requires more trials and computational power than traditional statistical models [136, 222]. Machine learning models are also vulnerable to ‘overfitting’, where they may show high accuracy rates for the training data used, but perform poorly when predicting out-of-sample values. Overfitting can be solved in a number of ways, such as by splitting a dataset into ‘training’ and ‘testing’ data, or by using out-of-sample data to test machine-learning models [67, 225, 302].

In the literature reviewed presently, machine learning was used primarily to predict consumer self-reported preference or buying behaviour [1–4, 6, 23, 24, 31, 85, 87, 96, 98, 101–103, 139, 151, 178, 185, 185, 186, 186, 206, 210, 223, 254, 262, 263, 287, 294, 307, 308, 314]. However, it has yet to be demonstrated how machine learning can be used to improve advertisement or product designs. For example, a machine learning approach could be used to suggest the shape or colour to use on product packaging. The integration of EEG machine learning methods with developing technologies such as VR headsets should also be investigated further (Fortunato 2014), as well as the use of ‘live’ machine-learning, which can actively update stimuli while a consumer is viewing them based on their patterns of brain activation (Robaina-Calderin 2021; Fortunato 2014).

## Conclusion

The literature summarised in the present systematic review highlighted the effectiveness of FAA and the LPP as measures of consumer preference and pointed to the importance of machine learning to tackle problems of consistency and replicability existent in the current literature. It is recommended that in future research, investigators use LPP and FAA effects when investigating customer preference and only use other EEG components to investigate other specifically associated effects (e.g., memory encoding, attentional orienting). Further, the use of machine learning is encouraged in future research to improve the replicability of EEG measures of customer preference, and the scope of machine-learning should be expanded.

## Data Availability

The search terms and inclusion/exclusion criteria required for replicability of the present research are included in the manuscript text.

## Abbreviations

DNN: Deep Neural Network
EEG: Electroencephalography
ERP: Event-related potential
ET: Eye-tracking
fMRI: Functional magnetic resonance imaging
fNIRS: Functional near-infrared spectroscopy
GSR: Galvanic skin response
HRV: Heart-rate variability
ICA: Independent component analysis
FAA: Frontal alpha asymmetry
FRN: Feedback related negativity
LPC: Late positive complex
LPP: Late positive potential
MMN: Mismatched negativity
PD: Pupil dilation
PET: Positron emission topography
PSW: Positive slow wave
TF: Time–frequency
